# Manifestation of Clinical Categories of Ocular Graft-versus-Host Disease

**DOI:** 10.1155/2018/6430953

**Published:** 2018-08-08

**Authors:** Yuan Qiu, Jing Hong, Rongmei Peng

**Affiliations:** Department of Ophthalmology, Peking University Third Hospital, Beijing, China

## Abstract

This study aims at improving the understanding of the subjective symptoms and signs of two different clinical categories of ocular graft-versus-host disease. After reviewing and screening 193 posthematopoietic stem cell transplantation (HSCT) patients of Peking University Third Hospital, we enrolled 148 (21 acute ocular GVHD, 127 chronic ocular GVHD). Patients' subjective symptoms, ocular parameters, and typical ocular signs were collected and evaluated at the same visit. Classic acute ocular GVHD patients had variable levels of conjunctival involvement but few had keratopathy; increased mucus secretion (21 of 21, 100.0%), red eye (19 of 21, 90.5%), and lacrimation (11 of 21, 52.4%) were the characteristic symptoms. The classic chronic ocular group had severe eye dryness and further corneal lesions, including filamentary keratitis, corneal ulcer, and corneal vascularization. Eye dryness (115 of 127, 90.6%), increased fibrous secretion (53 of 127, 41.7%), photophobia (50 of 127, 39.4%), and alacrimia (45 of 127, 35.4%) were the most common symptoms. Although 44.1% (56 of 127) of these patients had a history of acute ocular GVHD episodes, most were overlooked, so they did not receive stepwise evaluation and treatment. Management of ocular GVHD is very challenging and requires cooperation among disciplines.

## 1. Introduction

Allogeneic hematopoietic stem cell transplantation (allo-HSCT) is a potentially curative treatment for a variety of hematologic malignancies, and indications for HSCT could expand to other blood disorders, such as aplastic anemia, sickle cell disease, and immune disorders [1–3]. More than 25,000 HSCT procedures are performed annually, and the number of transplants and survival rates are increasing worldwide [4, 5].

Graft-versus-host disease (GVHD), an immune-mediated disease caused by complex interactions between donor and recipient immune systems, is a leading cause of morbidity and mortality following HSCT [4, 6, 7]. The 2014 National Institutes of Health Consensus recognized 2 principal categories of GVHD (acute and chronic) according to clinical features rather than the temporal relationship to the time of transplantation. Acute GVHD, which is stimulated by damaged recipient tissue and amplified by donor t-cells, includes the classic manifestations of erythema, maculopapular rash, nausea, vomiting, anorexia, profuse diarrhea, ileus, and cholestatic liver disease. Broad categories of this type of GVHD include classic and late-onset acute GVHD, which occurs within 100 days after transplantation or donor lymphocyte infusion. Chronic GVHD, related to thymic damage and impaired negative selection of autoreactive t-cells, is diagnosed according to at least one diagnostic manifestation or at least one distinctive manifestation plus a pertinent biopsy, laboratory, or other tests (e.g., Schirmer's test) in each organ [6, 8]. The simultaneous presence of features of acute GVHD in patients with chronic GVHD is defined as an overlap syndrome and is classified as a subset of chronic GVHD.

Ocular manifestations can be found in more than 60% of GVHD patients [9]. Dry eye is the most common symptom of GVHD; other distinctive manifestations of chronic ocular GVHD include gritty, cicatricial conjunctivitis, keratoconjunctivitis sicca, and confluent areas of punctate keratopathy [8, 10]. However, these symptoms are inadequate to diagnose ocular GVHD, and reports of other representative subjective symptoms and signs of ocular GVHD are limited. Thus, physicians are challenged by early recognition and referral of ocular GVHD.

The purpose of this study is to describe, analyze, and compare the characteristics of ocular manifestations of a large cohort of patients with a diagnosis of either acute or chronic ocular GVHD.

## 2. Materials and Methods

Records of 193 post-HSCT patients who visited Peking University Third Hospital Cornea and Ocular Surface Disease Specialist Clinic from July 2015 to July 2017 were reviewed. Patients who met the following criteria were eligible for the study: (1) diagnosed with ocular GVHD; (2) first visit to ophthalmology clinic; and (3) had not received topical immunosuppressant treatment. Criteria for acute ocular GVHD: (1) recent eye discomfort; (2) classic acute GVHD with skin, GI, or liver involvement; (3) without classic histological or clinical signs of chronic GVHD; and (4) no evidence of infection. Criteria for chronic ocular GVHD: (1) new ocular sicca documented by low Schirmer's test with a mean value of ≤5 mm at 5 minutes or (2) a new onset of keratoconjunctivitis sicca detected by slit lamp exam with mean Schirmer's test values of 6 to 10 mm [6].

We excluded patients with the following criteria: (1) signs of infection, glaucoma, retinopathy, allergy or other immune diseases; (2) with incomplete medical records; or (3) unable to be followed and interviewed in the clinic. The study was approved by the Peking University Third Hospital Medical Science Research Ethics Committee (protocol number: M2017275) and conducted in accordance with the Helsinki Declaration; all participants or their guardians provided written consent.

### 2.1. Outcome Measures

1. Data were collected on patients' demographic and transplant characteristics, such as age, gender, primary diseases for which HSCT was performed, donor-to-recipient information, systemic GVHD, and conditioning regimens.
2. *Patients' major symptoms*: patients' symptoms were evaluated with a special designed questionnaire which included 11 common subjective ocular discomforts (they were increased eye discharge, eye dryness, red eye, foreign body sensation, fluctuating vision, photophobia, burning sensation, lacrimation, alacrimia, edema of eyelids or conjunctiva, and ocular fatigue) at their first visit. The severity of each symptom was graded on a scale of 0 to 4, where 0 indicated none of the time; 1, some of the time; 2, half of the time; 3, most of the time; and 4, all of the time. Patients were asked to grade each symptom according to their present and previous initial discomfort feelings, respectively. We chose symptoms scored ≥3 as major ones for each patient in the study. The questionnaire also included essential time intervals (e.g., latency, i.e., interval from transplant to initial ocular symptoms, time interval between initial symptoms and first visit, and interval between transplant and first visit).
3. *Objective ocular examinations*: during each patient's first visit to the ophthalmology clinic, we collected the following ocular parameters: (1) best observed vision acuity (measured in logMAR); (2) intraocular pressure (measured with noncontact tonometer); (3) corneal fluorescein staining score (following the consensus on clinical diagnosis and treatment of dry eye in China, cornea was divided into four quadrants; then for each quadrant, no corneal epithelium stained scored 0, 1–30 stained points scored 1, >30 stained points without fusion scored 2, and punctate keratopathy fusion and/or corneal filiform paraphyte and/or corneal ulcer scored 3; final score for each eye is the sum of each quadrant's score) [11]; (4) fluorescein tear film break-up time (TBUT); (5) Schirmer's test score without anesthesia; and (6) conjunctival disease grade (acute GVHD: conjunctival hyperemia, stage I; hyperemia with chemosis and/or serosanguineous exudates, stage II; pseudomembranous conjunctivitis, stage III; and pseudomembranous conjunctivitis with corneal epithelial sloughing, stage IV) [12].

### 2.2. Statistical Analysis

Data were summarized as mean ± SD or as counts and proportions. We compared demographic, transplant characteristics, and objective ocular examination parameters of chronic and acute GVHD patients using independent sample *T*-test and Mann–Whitney *U* test. The chi-square test was used to compare frequencies of different symptoms between the two groups. A two-sided *P* value less than 0.05 was considered statistically significant.

## 3. Results

### 3.1. Patients' Demographics

**Figure 1:**
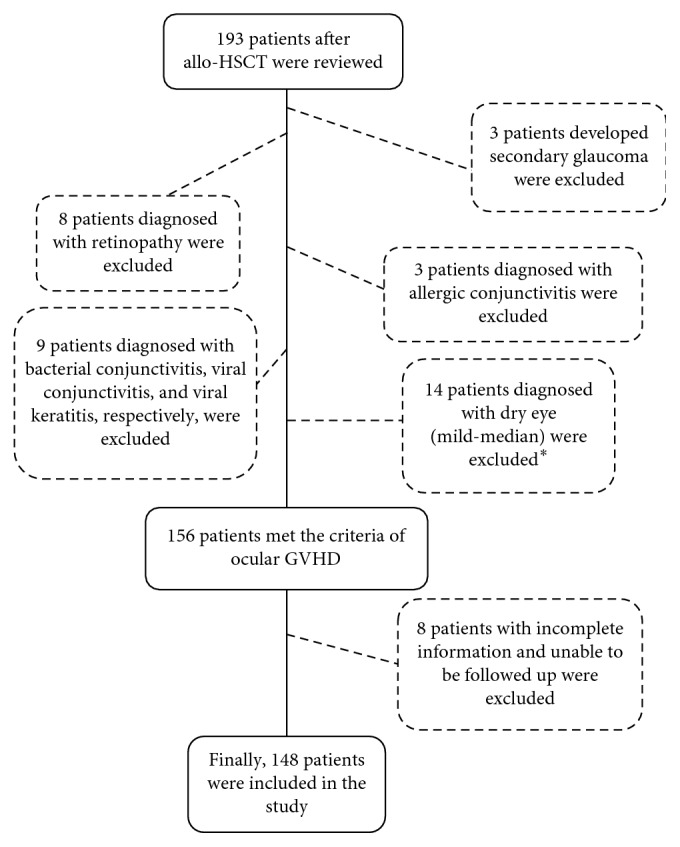
Flow diagram of patient selection. ^*∗*^These patients reported onset of dry eye symptoms, including irritation, burning sensation, dryness, or foreign body sensation that required treatment with frequent topical lubricants. However, signs of ocular surface abnormalities including decreased Schirmer's test (≤5 mm), tear break-up time (≤5 seconds), presence of conjunctivitis, and corneal fluorescein staining were absent.

Records of 193 consecutive post-allo-HSCT patients examined in the Department of Ophthalmology, Peking University Third Hospital, Beijing, were reviewed, and after screening, 148 patients were enrolled in the study (Figure 1). According to diagnostic criteria described above, patients were categorized into acute (21 of 148, 14.2%) or chronic (127 of 148, 85.8%) ocular GVHD groups. The demographics and transplant characteristics, shown in Table 1, were similar between the two groups (*P* > 0.05).

### 3.2. Subjective Symptom Questionnaire

#### 3.2.1. Acute Ocular GVHD Group

In our study, 21 patients were diagnosed with acute ocular GVHD. Mean latency from transplant to initial ocular symptoms was 8.0 ± 5.0 months; mean interval between transplantation and first visit was 8.6 ± 5.4 months. All patients, 21 of 21 (100%), visited ophthalmologists within 2 months of sensing initial ocular discomfort. The ocular symptoms reported at first visits were similar to their initial symptoms.

**Figure 2:**
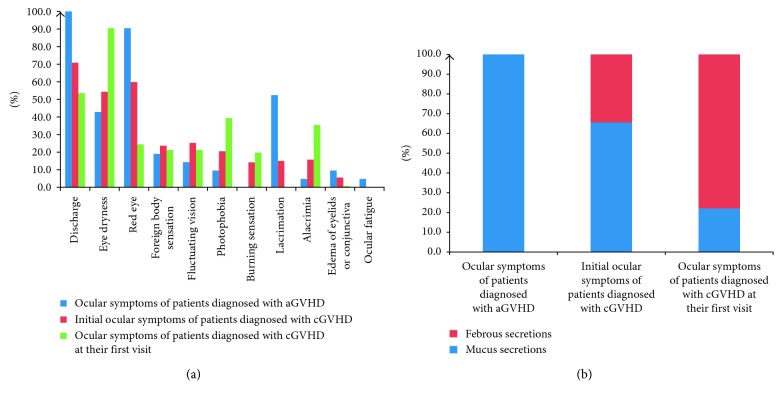
Symptoms of different clinical ocular GVHD categories. (a) Frequencies of different subjective symptoms. (b) Secretion compositions of different ocular disease GVHD categories. cGVHD = chronic graft-versus-host disease; aGVHD = acute graft-versus-host disease.

**Figure 3:**
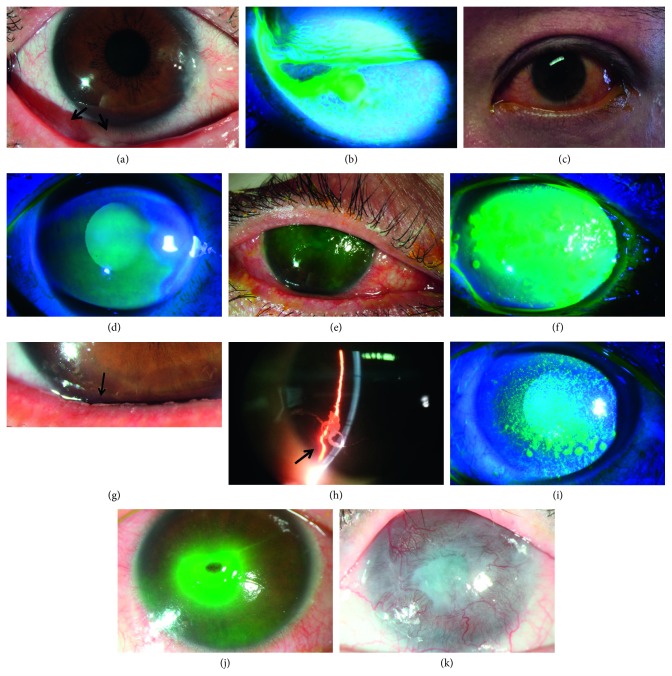
Typical ocular signs of ocular GVHD. (a)–(f) Signs of acute ocular GVHD and differential diagnosis: (a) mucus secretion (arrow) and conjunctival injection of acute ocular GVHD; (b) mucus secretion using fluorescein dye and visualized under a cobalt blue light; (c) and (d) conjunctiva and cornea signs of mild acute ocular GVHD; (e) and (f) conjunctiva and cornea signs of a post allo-HSCT patient who complained of red eye, lacrimation, and increased eye discharge and was diagnosed with viral corneal dermatitis eventually. (g)–(k) Signs and complications of chronic ocular GVHD: (g) and (h) typical fibrous secretion (arrow) of chronic ocular GVHD; (i) filamentary keratitis; (j) corneal perforation; and (k) corneal vascularization.

This group reported increased eye discharge (21 of 21, 100.0%), red eye (19 of 21, 90.5%), lacrimation (11 of 21, 52.4%), and eye dryness (9 of 21, 42.9%) most often. (Frequencies of each symptom are shown in Figure 2). Notably, all patients in this group described their eye discharge as clear and thick mucus secretions. Typical secretions of acute GVHD are shown in Figures 3 and 3.

#### 3.2.2. Chronic Ocular GVHD Group

Chronic ocular GVHD was diagnosed in 127 patients. Mean latency from transplant to initial ocular symptoms was 10.7 ± 9.1 months (similar to that of acute ocular GVHD, *P*=0.091). However, mean interval between initial discomfort and first visit was 9.9 ± 14.1 months, and the interval between onset of initial ocular discomfort and first ophthalmologist visit was >2 months for 82 of 127 (64.6%) and >6 months for 59 patients (46.5%).

The symptoms often reported at the first visit were eye dryness (115 of 127, 90.6%), increased eye discharge (68 of 127, 53.5%), photophobia (50 of 127, 39.4%), and alacrimia (45 of 127, 35.4%). Among patients with increased eye discharge, 77.9% (53 of 68) described discharge as white and stringy fibrous secretions, and the other 22.1% (15 of 68) reported mucus secretions. Typical fibrous secretions are shown in Figures 3 and 3.

Among initial symptoms recalled at the first visit, increased eye discharge (90 of 127, 70.9%), red eye (76 of 127, 59.8%), eye dryness (69 of 127, 54.3%), and fluctuating vision (32 of 127, 25.2%) were most common. In addition, 44.1% (56 of 127) of patients reported symptoms (mucus ocular secretions, red eye and systemic symptoms) that were similar to typical acute ocular GVHD manifestations in our study, and they could recall definite events related to the occurrence of initial ocular symptoms. The events included increased intensity of conditioning regimens (35 of 52, 67.3%), treatment with interferon (IFN)-a2b (10 of 52, 19.2%), retransplant or reinfusion of donor lymphocyte (5 of 52, 9.6%), fatigue (1 of 52, 1.9%), and pulmonary infection (1 of 52, 1.9%). Ocular manifestations improved with intensive conditioning immunosuppressant, and these patients were followed in oncology or internal medicine departments until severe ocular complications occurred or symptoms became unbearable.

Frequencies of subjective symptoms of different clinical ocular GVHD categories are shown in Figure 2(a). In Figure 2(b), we analyzed secretion composition differences between GVHD categories. The frequencies of characteristic symptoms (increased mucus secretion, red eye, and lacrimation) in the acute ocular GVHD group differed significantly from those inherent to the chronic ocular GVHD group (eye dryness, increased fibrous secretion, alacrimia, and photophobia) (*P* < 0.05).

#### 3.2.3. Objective Ocular Examinations

Best corrected visual acuities and intraocular pressures were all within normal limits and were similar between the two groups. Patients in the acute GVHD group had different levels of conjunctival involvement but few had keratopathy (corneal fluorescein staining score 2.5 ± 3.8) (Figures 3 and 3). 59.5 percent (25 of 42) of eyes were in stage I of conjunctival disease, and 21.4 percent (9 of 42) of eyes (stage III and stage IV) appeared to have severe conjunctival and corneal involvement of pseudomembranous conjunctivitis, corneal epithelial sloughing, and limbus damage. However, the chronic group had severe eye dryness and further corneal lesions: 30 (11.8%) eyes developed filamentary keratitis (Figure 3), 20 (7.9%) eyes developed corneal ulcer, 6 (2.4%) eyes developed spontaneous corneal perforation (Figure 3), and 2 (0.8%) eyes developed corneal vascularization (Figure 3). Corneal fluorescein staining, TBUT, and Schirmer's tear secretion score were also significantly different from the acute GVHD group (*P* < 0.05) (Table 2).

## 4. Discussion

### 4.1. Manifestations of Acute Ocular GVHD

According to previous studies, the prevalence of ocular involvement in post-HSCT patients is approximately 30%–40%; acute GVHD only accounts for 1% of this [13, 14]. Studies of acute ocular GVHD are limited. Jabs et al. [12] described distinct-appearing conjunctivitis as an acute GVHD involvement of the conjunctiva and formulated a clinical-staging system from mild conjunctival hyperemia to severe pseudomembranous conjunctivitis with corneal epithelial sloughing (see disease-grading details in *Materials and Methods*). Uchino et al. [15] reported a juvenile case of pseudomembranous conjunctivitis accompanied with corneal ulcer (stage IV) and corneal epithelial defects, which eventually disappeared after pseudomembrane excision. Inflammatory cells, including macrophages, neutrophils, T cells, and natural killer cells that accumulate in the conjunctiva in acute ocular GVHD promote the release of cytokines (tumor necrosis factor (TNF)-*α*, interleukin (IL)-6, etc.) that resulted in further corneal epithelial defects [8, 15]. Thus, it is our belief that conjunctival involvement is the early and primary manifestation of acute ocular GVHD.

In our study, patients with acute ocular GVHD all demonstrated acute conjunctival inflammation but fewer had lacrimal secretion dysfunction and corneal lesions. The most characteristic acute ocular GVHD manifestation was increased mucus eye secretions. We found corresponding signs in each affected eye. Red eye and lacrimation were also frequent in this group. In our study, the majority of patients with acute ocular GVHD had mild-moderate involvement and often could be cured with intensive conditioning immunosuppressant. However, it is advantageous to follow these patients closely because patients developed pseudomembrane formation and/or corneal epithelium loss (stage III-IV) that indicated a severe systemic involvement by GVHD as well as decreased survival rate [4, 12]. Moreover, acute ocular involvement is a predecessor of chronic ocular GVHD [16]. It is also important and necessary to exclude the diagnoses of anterior segment infections, including viral keratitis, viral conjunctivitis, and bacterial conjunctivitis that could occur in post-HSCT patients (Figures 1 and 3).

Although eye dryness was frequently reported, Schirmer's test, TBUT, and corneal fluorescein staining scores were almost always within normal limits. Perhaps, poor reading habits, underlying diseases, and conditioning regimens led to dry eye problems.

### 4.2. Manifestations of Chronic Ocular GVHD

Dry eye is the most common manifestation of chronic ocular GVHD. The National Institutes of Health censensus document defines dry eye and corresponding ocular signs as distinctive manifestations of chronic ocular GVHD. An ocular distinctive manifestation with impaired Schirmer's test is sufficient for the diagnosis of the disease [6]. The international chronic ocular consensus group strongly recommended dry eye disease as a diagnostic clinical entity for chronic GVHD [4]. In our study, ocular chronic GVHD severe eye dryness predominated and was accompanied by corresponding serious ocular signs, including corneal fluorescein staining (6.7 ± 4.8), decreased TBUT (2.1 ± 1.8 seconds), and Schirmer's tear secretion (3.1 ± 2.4 mm). Those findings are consistent with previous research and consensus findings. Increased fibrous eye secretions, photophobia, and alacrimia were common complaints in this group. Besides, photophobia, burning sensation, foreign body sensation, and fluctuating vision indicated further corneal lesion, for example, punctate keratopathy fusion, filamentary keratitis, corneal ulcer, and corneal perforation occurred in 10%–40% eyes of this group. In our study, 22.8% of chronic ocular GVHD patients developed severe ocular complications; spontaneous corneal perforation, corneal vascularization, and even endophthalmitis have been reported before [17–19]. Moreover, chronic ocular GVHD greatly impairs patient's vision-related quality of life and affects their educational level, job position, and underlying disease [20, 21].

However, we also found that 44.1% (56 of 127) of chronic ocular GVHD patients had a definite history of acute ocular GVHD episode. Ocular symptoms that were mild initially were ignored or only treated with artificial tears. Over time, manifestations progressed and patients were finally referred to ophthalmologists when severe ocular complications arose or symptoms became unbearable. These patients might have missed the best opportunity for prophylaxis and treatment to restore lacrimal secretion function. Had patients reported mild discomfort, we could have detected the ocular surface changes of chronic ocular GVHD directly and, thus, given comprehensive treatments with early intervention and regular follow-up.

Our primary limitation is the retrospective nature of the study; patients' recall of initial subjective symptoms on the questionnaire may also be inaccurate. Ocular GVHD patients always struggle with visiting an ophthalmological clinic promptly due to burdens of relapses of underlying disease, systemic GVHD, and infections. Ophthalmologists and physicians are challenged to recognize, diagnose, and evaluate ocular GVHD, and even more so to understand progression of the disease. We tried to report accurate results. Few essential records were missing, and we confirmed data from medical records with patients or their families through face-to-face interviews. Certainly, further large, long-term, prospective clinical studies are necessary to better describe this disease accurately.

In summary, acute conjunctivitis with increased mucus eye secretions, red eye, and lacrimation indicates a high probability of acute ocular GVHD if differentiated from infections. Chronic ocular GVHD is characterized by severe dry eye and further corneal lesions, including filamentary keratitis, corneal ulcer, and corneal vascularization. After diagnosis, it is not difficult to determine ocular GVHD subsets like classic acute or chronic GVHD, overlap syndrome, and late-onset acute GVHD. At an early stage, most acute ocular GVHD and chronic GVHD cases are overlooked and therefore do not undergo stepwise evaluation or treatment, which eventually leads to serious ocular complications and poor quality of life. The ultimate goal is to offer better, early prophylaxis and treatment of the discussed complications. Ocular GVHD or dry eye syndrome of chronic GVHD should be referred to ophthalmologists who can then intervene early to evaluate, diagnose, and treat the corresponding conditions.

## Figures and Tables

**Table 1:** Demographics and transplant characteristic by patient groups.

	Acute GVHD	Chronic GVHD
	(*n*=21, eyes = 42)	(*n*=127, eyes = 254)
Age (yrs.), mean ± SD	29 ± 12	28 ± 11
*P* (independent sample *T*-test)	0.704	

Gender		
Male	13 (61.9%)	79 (62.2%)
Female	8 (38.1%)	48 (37.8%)
*P* (chi-square test)	0.979	

Primary disease		
Acute lymphoid leukemia	2 (9.5%)	38 (29.9%)
Acute myeloid leukemia	9 (42.9%)	48 (37.8%)
Myelodysplastic syndrome	6 (28.6%)	31 (24.4%)
Chronic myeloid leukemia	1 (4.8%)	5 (3.9%)
Non-Hodgkin's lymphoma	0 (0.0%)	3 (2.4%)
Hodgkin's lymphoma	1 (4.8%)	0 (0.0%)
Aplastic anemia	2 (9.5%)	2 (1.6%)

Donor-recipient gender disparity		
Female donor to male recipient	8 (38.1%)	36 (28.3%)
Male donor to female recipient	4 (19.0%)	33 (26.0%)

Essential time points		
Interval from transplant to initial ocular symptoms (months), mean ± SD	8.0 ± 5.0	10.7 ± 9.1
*P* (Mann–Whitney *U* test)	0.091	
Interval between initial symptoms and first visit (months), mean ± SD	0.6 ± 0.7	9.9 ± 14.1
*P* (Mann–Whitney *U* test)	<0.001	
Interval between transplant and first visit (months), mean ± SD	8.6 ± 5.4	20.4 ± 17.7
*P* (Mann–Whitney *U* test)	<0.001	

GVHD = graft-versus-host disease.

**Table 2:** Outcomes of objective ocular examinations of two groups.

Outcomes	Acute GVHD	Chronic GVHD
	(*n*=21, eyes = 42)	(*n*=127, eyes = 254)
Best corrected visual acuity (logMAR)		
Mean ± SD	0.23 ± 0.24	0.24 ± 0.27
*Z*	−0.165	
*P* (Mann–Whitney *U* test)	0.869	

Intraocular pressure (mmHg)		
Mean ± SD	13.42 ± 2.04	14.11 ± 2.70
*Z*	−1.566	
*P* (Mann–Whitney *U* test)	0.117	

Corneal fluorescein staining		
Mean ± SD	2.5 ± 3.8	5.8 ± 3.9
*Z*	−5.726	
*P* (Mann–Whitney *U* test)	<0.001	

Schirmer tear secretion score (mm)		
Mean ± SD	9.0 ± 3.6	2.8 ± 2.0
*Z*	−9.623	
*P* (Mann–Whitney *U* test)	<0.001	

Fluorescein tear film break-up time (seconds)		
Mean ± SD	5.7 ± 3.6	3.2 ± 2.7
*Z*	−4.054	
*P* (Mann–Whitney *U* test)	<0.001	

GVHD = graft-versus-host disease.

## Data Availability

The data used to support the findings of this study are available from the corresponding author upon request.
